# A polymorphic inframe deletion in the ODR-10 extracellular loop 2 abolishes diacetyl sensing

**DOI:** 10.17912/micropub.biology.001722

**Published:** 2025-07-26

**Authors:** Aatira Mehraj, Rémy Mimbré, Katie Pelletier, Varsha Singh, Marie-Anne Félix

**Affiliations:** 1 Institut de Biologie de l'École Normale Supérieure, Paris, Île-de-France, France; 2 Department of Developmental Biology and Genetics, Indian Institute of Science, Bengaluru, India; 3 Division of Molecular Microbiology, School of Life Sciences, University of Dundee, UK

## Abstract

The *C. elegans* wild strain DL226 carries a 30 bp inframe deletion in the *odr-10* gene coding for the diacetyl olfactory receptor. DL226 animals are defective for attraction to diacetyl but not to pyrrole, an unrelated odorant also sensed by AWA neurons. Using genome editing in the N2 background, we show that this inframe deletion is causal for the defect in diacetyl sensing. The deletion specifically removes the predicted ligand-binding extracellular loop 2 (ECL2).

**Figure 1. A 30 bp deletion in the odr-10 extracellular loop 2 domain abolishes diacetyl sensing in the wild C. elegans strain DL226:**
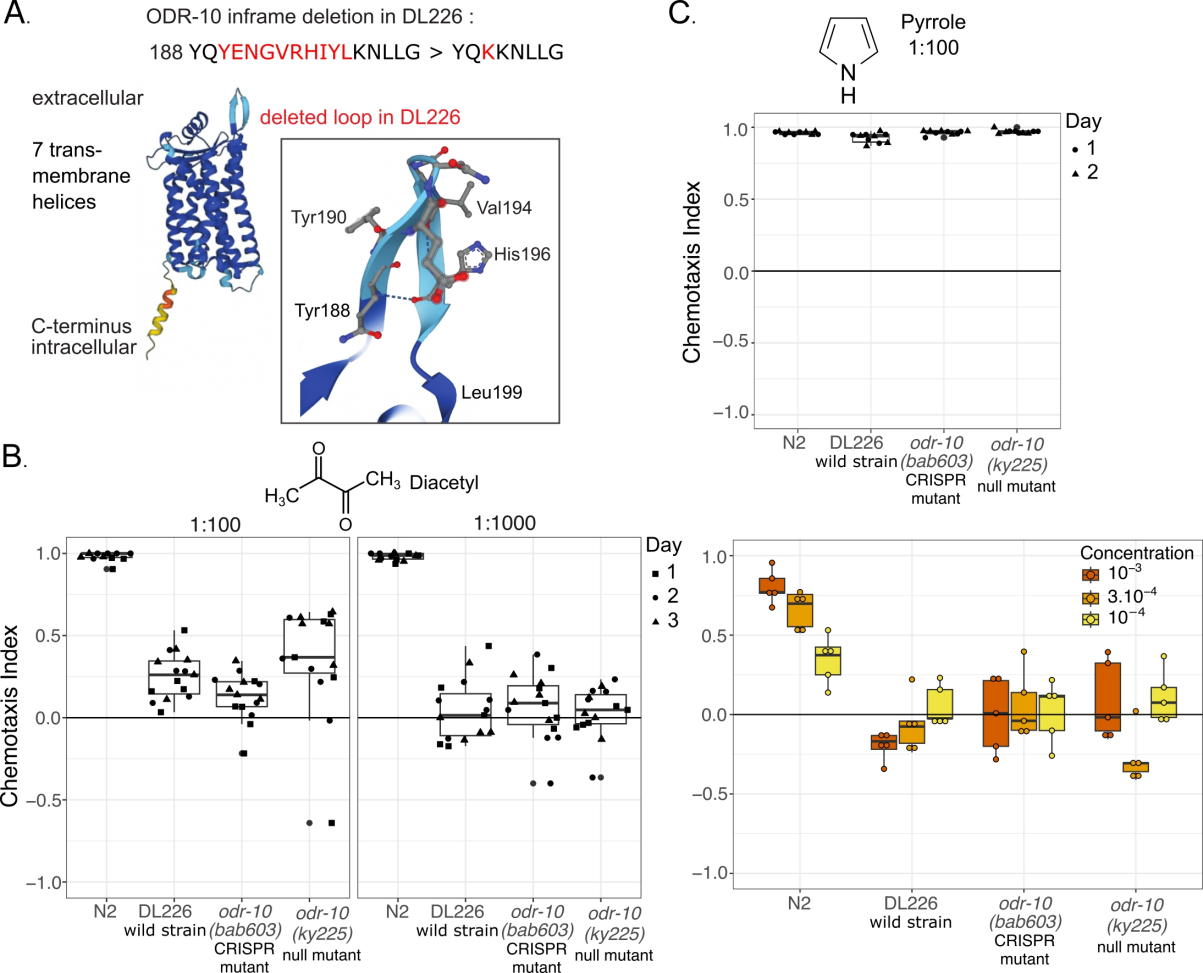
**(A)** Change in the predicted ODR-10 protein sequence in DL226 , with the altered amino-acids highlighted in red in the primary sequence. Below is shown the predicted protein structure using Alphafold where the colors correspond to estimated quality of the prediction (decreasing from dark blue to light blue, yellow and red). The deleted extracellular loop 2 is indicated on the top right and magnified in the box. Tyr190 and Leu199 are the first and last amino-acids of the deleted segment, respectively. Tyr188 is the first of the predicted beta-sheet. Some other amino-acids bulging out on the predicted structure are highlighted. **(B)** Olfaction tests using diacetyl. The graph on the left (1:100) corresponds to three experimental blocks and the graph in the center (1:1000) to three other experimental blocks and are thus plotted separately. Shapes of datapoints within each graph correspond to blocks. The graph on the right corresponds to a single experiment at three concentrations. Statistics are provided in the text and Extended Data Table. **(C)** Olfaction test using 1:100 pyrrole, conducted in two experimental blocks indicated by datapoint shapes.

## Description

The first *C. elegans* G-protein coupled olfactory receptor to be identified through a genetic screen *, odr-10* , is expressed in AWA neurons and confers attraction to diacetyl (Sengupta et al., 1996; Troemel et al., 1997). Its ectopic expression in AWB chemosensory neurons is sufficient to confer diacetyl repulsion (Troemel et al., 1997). The *C. elegans* genome contains thousand such G-protein coupled receptors (GPCRs), evolving by duplications, divergence and pseudogenization (Robertson and Thomas, 2006). In natural populations of *C. elegans* , many olfactory receptors are potentially under selection for the capacity to detect relevant odors in specific environments, as found in *Drosophila* for ionotropic olfactory receptors (e.g. Benton, 2022; Pellegrino et al., 2011; Prieto-Godino et al., 2017). Of note, a non-sense polymorphism in a putative diacetyl receptor was found by association mapping using single-nucleotide polymorphisms and olfaction tests in humans, and diacetyl binding of the corresponding protein was confirmed using in vitro assays (Trimmer et al., 2019).

We examined polymorphisms in the *C. elegans odr-10* gene using genome-wide polymorphism data in CeNDR (Cook et al., 2017). Unlike many other GPCR genes, *odr-10* is located in a genomic region that does not contain hyperdivergent genotypes (Lee et al., 2021). The gene accordingly displays few non-synonymous polymorphisms and no nonsense mutation. The most striking polymorphism is a 30 bp inframe deletion in the DL226 strain, isolated from Oregon, USA ( Figure 1A ). We further focus on this indel polymorphism.

We tested the DL226 wild strain for attraction to diacetyl ( Figure 1B ) and found a significant reduction compared to the N2 strain in attraction towards a diacetyl spot at a 1:1000 dilution (log odds ratio = 4.81, 95% CI = 3.82 – 5.80, *z* = 12.5, *p* < 10 ^-4^ ) and at a 1:100 diacetyl dilution (log odds ratio = 4.11, 95% CI = 3.19 – 5.02, *z* = 11.6, *p* < 10 ^-4^ ). We then tested whether DL226 was sensitive to another odor sensed by the AWA neurons, pyrrole, and found no significant effect (log odds ratio = 0.62, 95% CI = -0.24 – 1.48, *z* = 1.86, *p* = 0.25) ( Figure 1C ). This result showed that DL226 animals are able to respond in the olfaction test and are not generally defective for olfaction by the AWA neuron.

Using CRISPR/Cas9 genome editing, we obtained the 30 bp deletion allele in the N2 background (allele *bab603* ) and tested it in parallel with DL226 and a strain carrying the null deletion allele *odr-10 ( ky225 )* (Sengupta et al., 1996). Our results showed that the deletion alone could explain the DL226 diacetyl phenotype. This deletion also mimicked the null allele for diacetyl sensing as there was no significant difference in attraction between strains at 1:100 (log odds ratio = -0.42, 95% CI = -0.86 – 0.018, *z* = -2.46, *p* = 0.066) and 1:1000 (log odds ratio = 0.067, 95% CI = -0.18 – 0.31, *z* = 0.11, *p* = 0.89) diacetyl dilutions ( Figure 1B ). The variation in diacetyl sensing between N2 and DL226 is thus possibly monogenic and explained by this indel polymorphism.

The *odr-10* deletion in DL226 remarkably removes amino-acids in the extracellular loop 2 (ECL2) of the GPCR between trans-membrane domains 3 and 4. ECL2 is the largest extracellular loop, usually involved in binding odorants in GPCRs (Wheatley et al., 2012; Yu et al., 2022). In the predicted protein structure using AlphaFold, the deleted amino-acids strikingly correspond to a small putative beta-sheet fold predicted to extend out of the protein ( Figure 1A ).

The 30 bp inframe deletion is derived, as it is not found in the close duplicate *str-112* (Robertson, 2001), nor in the outgroup *C. briggsae* orthologs. Th *e str-112* gene is a close duplicate of *odr-10* (Robertson, 2001), also expressed in AWA (Chen et al., 2014). The STR-112 receptor differs from ODR-10 in ECL2, including Val194Glu, and it is not redundant with ODR-10 .

Overall, the *odr-10* gene is highly conserved among *C. elegans* strains, suggesting strong stabilizing selection. The deletion studied here was so far only found in DL226 (Cook et al., 2017). It is possible that local conditions favored an absence of attraction to diacetyl in the populations where DL226 comes from. Alternatively, it may correspond to a rare deleterious mutation; however, it appears unlikely that a random deleterious mutation would have inactivated the receptor by an inframe deletion of this small extracellular domain, rather than a frameshift, stop codon or inframe deletion in other parts of the protein. Also, if the small indel behaved as a full knockout, it would have been likely to accumulate further mutations making it a pseudogene, like many *C. elegans* GPCRs (Robertson and Thomas, 2006) - a caveat being that the mutation may be too recent. The puzzle remains of whether the protein carrying this partial deletion without pseudogenization can bind another odorant or have a different activity.

## Methods

**Strains and culture**

All *C. elegans* strains used in this study were maintained as hermaphrodites at 20°C on Nematode Growth Medium (NGM) poured into Petri plates and seeded with *E. coli* OP50 , with prior bleaching to remove possible contamination micro-organisms (Stiernagle, 2006). The strains used in this study are listed in the table below:

**Table:** 

Strain	Genotype	Source
N2	reference strain	Paul Sternberg
DL226	wild isolate	C. Hilburn, Dee Denver, Robyn Tanny
MCP603	*odr-10 ( bab603 ) *	this work
CX3410	*odr-10 ( ky225 ) *	CGC

The *odr-10 ( ky225 )* allele is a partial deletion of the locus obtained using transposition and imprecise excision of the *Tc1* transposon (Sengupta et al., 1996).

**Natural deletion and CRISPR/Cas9 genome editing**

The N2 allele at the *odr-10* locus include *s* the sequence TCCAAGCAAGTTTTTgaggtagatatgccttactccgttttcgtaTTGGTACTGAAAGTATA (Sternberg et al. 2024). In the DL226 wild strain, the 30 bp in small letters are deleted and repaired with addition of TTT, resulting in the inframe sequence TCCAAGCAAGTTTTTTTTTTGGTACTGAAAGTATA.

The *odr-10 ( bab603 )* allele reconstitutes the DL226 deletion in the N2 background and was obtained using CRISPR/Cas9 genome editing by the SegiCel platform (Lyon, France), according to methods in Dokshin et al. (2018). An injection mix with 0.25 µg/µL Cas9 protein (IDT), 3 pmol/µL of the duplex crRNA-MG048/tracrRNA, 110 ng/µL sODN oMG156 and 40 ng/µL pRF4 ( *rol-6* with a dominant mutation conferring a Roller phenotype). The replacement was screened using PCR primers oMG157-159 designed using WormBase (Sternberg et al. 2024) and checked using Sanger sequencing.

**Table:** 

Guide RNA CrMG048	ACCAATACGAAAACGGAGTA
Repair oMG173	acggaaaccatcaaactagatactttcagTACCAAaaaAAAAAg TTGCTTGGATGCTTTGTTCATTACTTTGTCATGgt
oMG157	TACCCGTGACAATGTGGGC
oMG158	TCGAGTCCCGGGTACAGAAA
oMG159	ACGAAAACGGAGTAAGGCAT

**Chemotaxis assay**

Chemotaxis assays were performed in 90 mm buffered agar plates (NaCl, 2% agar, 1 mM CaCl _2,_ 1 mM MgSO _4_ , 25 mM KPO _4_ buffer and 5 mg/ml Cholesterol), without bacteria. 2 µL of 1 M sodium azide were spotted on two opposite sides of the plate 0.5 cm away from the edge to immobilize the animals once they moved to one side. 2 µL of test and control (solvent) were then spotted near the sodium azide spot on opposite sides of the plate 1 cm away from the edge. The solvent for the chemicals used in this study is chloroform for both diacetyl and pyrrole. 70 h after a 4 h egg lay at 20°C. Gravid adult hermaphrodites were washed with S-Basal Buffer 3 times and approximately 60-80 worms were spotted in the center of the plate. The plates were covered with parafilm and incubated at 25°C for 3 hours. After the incubation, animals were counted on each side of the plate (i.e. near the control and the test) and elsewhere. The Chemotaxis Index (CI) was calculated for each replicate using the following equation:

Chemotaxis Index (CI) = (Animals on the test side – Animals on the control side)/ Total number of animals

The diacetyl tests were performed in several independent blocks (experiments) for each concentration of diacetyl. Detailed scorings are available as Extended Data Table.

**Statistics**

All statistics were performed using the R programing language (R Core Team, 2023). We wished to account for the possible effect of blocks (different experiments) and to use a test that had no requirement about normality of the residuals. Thus, probability of attraction to the odorant was estimated using a generalized linear mixed model with a binomial family distribution for the data using the *glmmTMB* package (v. 1.1.8) (Brooks et al., 2017). The observed number of animals in the test side and control side of the plate were modeled as a function of genotype and a random effect of genotype nested within block to account for block effects. Note that very few animals were not found on either side at the end of the experiments (see Extended Data Table). Estimated marginal means for each phenotype, with 95% confidence intervals, were calculated using the *emmeans* package (v. 1.8.9) (Lenth et al., 2023). Pairwise comparisons between genotypes as log-odds ratio were tested using contrasts from the emmeans package, with a Tukey adjustment of *p* -values to account for multiple testing. The R code is provided in the Extended Data file 'chemotaxis_assay.Rmd'. Detailed statistics are available in the Extended Data Table.

**Variation in results of the diacetyl chemotaxis assay**

We note that in previous experiments performed in the Singh laboratory in Bangalore (Siddiqui et al. 2024), DL226 displayed a positive chemotaxis index in the 0.7-0.8 range, even though it was significantly less attracted than N2 . We first thought that the results presented in the left and middle graphs of Figure 1B were explained by the age (20 years) of the diacetyl bottle in the Félix laboratory. We ordered a new bottle and obtained a different result on two different days (see Supplemental Dataset). A 10 ^-3^ dilution in some experiments produced no attraction (index close to 0; Figure 1B, right) from the *odr-10* mutants while on other days the median attraction index reached 0.5 (see Extended Data Table, sheet RM_diacetyl). The concentration range at which *odr-10* mutants fail to chemotact also varies between published articles (Sengupta, Chou, and Bargmann 1996; Ryan et al. 2014). We suspect that the dose-response range of both wild-type and mutant depends on many environmental factors that we do not control. A recent method article points to steps that could be sensitive (Cesar and Morud 2025).

This variability does not change the main conclusion that DL226 is less attracted to diacetyl than N2 and that the *odr-10* inframe deletion is a main causal polymorphism.

**Protein structure prediction**

We used the European Bioinformatics Institute server ( https://alphafold.ebi.ac.uk/ ) for a protein structure prediction based on Alphafold (Jumper et al., 2021).

## Data Availability

Description: Code used for statistical analysis.. Resource Type: Model. DOI: https://doi.org/10.22002/bszpz-kya58 Description: Extended Data Table with the raw counts of animals on the olfaction plates, additional experimental blocks, and statistics.. Resource Type: Dataset. DOI: https://doi.org/10.22002/zt0gm-y5z17
